# A case report of ultrasound-guided knee nerve pulse radiofrequency combined with platelet-rich plasma in the treatment of knee osteoarthritis

**DOI:** 10.1097/MD.0000000000027878

**Published:** 2021-12-23

**Authors:** Hui Jin, Hao Zuo, Rui Xu, Youbo Ji, Zhonghan Wang

**Affiliations:** a Department of Pain, The Second Hospital of Jilin University, Changchun, China; b Department of Endocrinology, Shanghai National Research Center for Endocrine and Metabolic Disease, State Key Laboratory of Medical Genomics, Shanghai Institute for Endocrine and Metabolic Disease, Ruijin Hospital, Shanghai Jiaotong University School of Medicine, Shanghai, PR China; c Department of Orthopedics, The Second Hospital of Jilin University, Changchun, Jilin, PR China.

**Keywords:** knee joint, osteoarthritis, platelet rich plasma, pulsed radiofrequency, ultrasound guidance

## Abstract

**Rationable:**

Knee osteoarthritis (KOA) is a disease characterized by noninflammatory degenerative changes of articular cartilage. The main clinical manifestations are joint pain and stiffness. Pulsed radiofrequency (PRF) is thought to treat pain by destroying nerve tissue and changing the physical characteristics of nerve tissue membrane.

**Patient concerns:**

The patients presents with joint pain and tenderness. Touching around the knee joint will induce pain and joint stiffness when the hand is pressed hard.

**Interventions:**

Four patients with knee osteoarthritis underwent pulsed radiofrequency thermocoagulation in the knee joint cavity under ultrasound guidance and injected 2 mL of 10 mg/mL platelet-rich plasma into the joint cavity once a week for a total of 4 times. Record the patient's Visual Analogue Scale (VAS) score and the degree of knee movement limitation before treatment, 1, 3, and 6 months after treatment.

**Diagnoses:**

Four patients with knee osteoarthritis.

**Outcomes:**

After treatment, the patient's VAS score improved, and the knee joint mobility function recovered well. Ultrasound-guided knee nerve pulse radiofrequency combined with intra-articular injection of platelet-rich plasma can effectively improve the knee joint function and reduce the pain of the patient. The clinical effect is significant, and it is worthy of clinical application.

## 1 Introduction

Knee osteoarthritis (KOA) is a disease characterized by noninflammatory degenerative changes of articular cartilage, and it is one of the major disabling diseases in the world. The main clinical manifestations are joint pain and stiffness, especially after exercise, which will have a greater impact on the patient's daily life. It is known that KOA is more common in people with obesity problems, and the prevalence of KOA increases sharply with age. With the aging of the population around the world and the increase in obesity, it is expected that the economic burden of personal and social health caused by KOA will become a major problem in the global health system.^[1]^

When KOA develops to the advanced stage, total knee arthroplasty is the currently accepted treatment method.^[2]^ A report pointed out that as many as 15% of total knee replacement patients may experience severe or extremely persistent pain 3 to 4 years after surgery.^[3]^ These patients often continue to require a higher level of utilization of medical resources. In addition, osteoarthritis patients undergoing total knee arthroplasty may experience a pain that is more difficult to diagnose and treat than patients who have not undergone knee arthroplasty.^[4,5]^ Therefore, in order to ensure the rationalization of medical resources and costs, conservative treatment is particularly important. The main purpose of conservative treatment is to reduce pain, improve function and quality of life, and limit disease progression. When considering various possible conservative treatment methods and uncertain factors based on insufficient evidence, there are inevitably some differences between clinical diagnosis and treatment guidelines. However, the best conservative treatment of knee osteoarthritis requires a combination of drug and non-drug treatment modes according to the individual needs of patients.^[6,7]^

Non-steroidal anti-inflammatory drugs mainly inhibit cyclooxygenase and reduce the production of inflammatory mediator prostaglandins, thereby producing anti-inflammatory effects. They are the first-line drugs for the treatment of KOA.^[8]^ However, long-term use can cause side effects such as gastrointestinal, cardiovascular and renal damage.^[9]^ If oral drugs are ineffective, intra-articular injections are the last nonsurgical treatment that can be the first choice.^[10,11]^ It can effectively reduce pain and is safer than oral medication.^[12]^ Platelet-rich plasma (PRP) is a platelet concentrate obtained from autologous whole blood after centrifugation. A variety of growth factors, coagulation factors, adhesion molecules, cytokines, chemokines and integrins are all present in platelets. Intra-articular injection has the potential to fill cartilage defects, promote cartilage repair, reduce symptoms of osteoarthritis and improve joint function.^[13]^

The knee nerves that innervate the knee joint include the obturator nerve, saphenous nerve, femoral nerve, common peroneal nerve, and tibial nerve. Knee nerve radiofrequency ablation (RFA) has been studied as an alternative nonsurgical treatment for chronic knee osteoarthritis pain, which increases function and relieves pain by damaging the nerves that innervate the painful tissue or by reducing the transmission of pain signals.^[14,15]^ Pulsed radiofrequency (PRF) provides pulse energy with a frequency of 1 to 8 hz, and the radio frequency within each pulse is 500 KHz. PRF is believed to be able to treat pain by destroying nerve tissue and changing the physical characteristics of nerve tissue membranes.^[16]^ Compared with radiofrequency ablation, its reliable advantage is that pulses will not increase the average temperature of the target tissue, so irreversible nerve tissue destruction will not occur. And PRF is safer when applied to nerves with motor and autonomic nerve fibers.^[17]^ Ultrasound guidance reduces the technical difficulty of locating nerves by visualizing the blood vessels that accompany the nerves and provides a relatively fast and non-invasive method that can directly see the knee nerves and peripheral vascular system.^[18]^

## 2 Patient concerns

The patients presents with joint pain and tenderness. Touching around the knee joint will induce pain and joint stiffness when the hand is pressed hard.

## 3 Diagnosis

A retrospective analysis of 4 patients in our hospital from August 2019 to June 2020, including 2 males and 2 females. Inclusion criteria: ① Age: 50 to 74 years old; ② Tenderness around the affected knee joint, mild to moderate swelling, increased pain when the knee joint is weight-bearing, and mildly limited joint functional activities; ③ X-rays suggest that the joint space of the affected knee joint is light The degree of narrowing; ④ The duration of knee joint pain and mobility disorders >3 months. Exclusion criteria: ① Knee joint pain related to radiculopathy; ② Previous severe mental illness; ③ Injection of glucocorticoid and sodium hyaluronate into the joint cavity within 3 months.

## 4 Methodology

**Figure 1:**
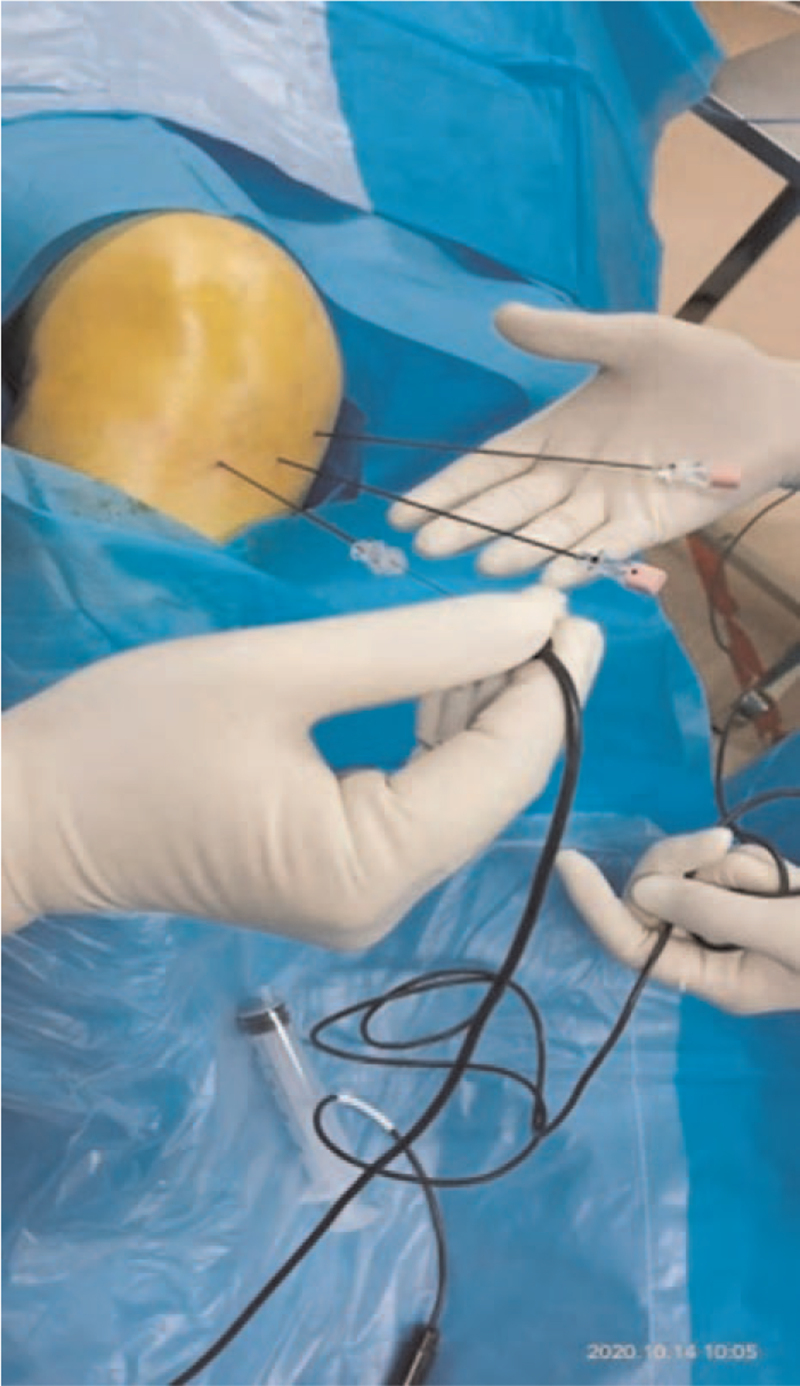
Radiofrequency treatment of knee joint.

All patients signed informed consent. The Ethics Committee of the Second Hospital of Jilin University approved the study. The patient was placed in the supine position, the knee joint was raised at a 30° angle, and ECG monitoring and low-flow oxygen inhalation were given. Under ultrasound monitoring, the upper medial, upper lateral and lower medial knee nerves were identified, which were mainly close to the arteries of the same name. The upper medial knee nerve and the upper lateral knee nerve surround the medial and lateral sides of the femoral shaft, respectively. The inferior medial knee nerve is distal to the tibial neck and medial epicondyle. Mark the tenderness points around the knees and eyes on both sides, and the distance between each point is 1 to 1.5 cm. After routine disinfection, 1 mL of 1% lidocaine plus 0.5% ropivacaine mixture was used for subcutaneous infiltration anesthesia until the bone surface. The radiofrequency needle enters the joint from both knees and eyes, and the target nerve is electrically stimulated at 50 Hz, 2 Hz, greater than 1.0 to 1.5 V, without causing abnormal feelings in the innervation area of the calf and muscle movement. It is recognized that the radiofrequency puncture needle is not close to the nerve. No damage to nerves. Subsequently, it is advocated to implement pulsed radio frequency, the parameters are 42°C, the duration is 120 second, the total heating time is 12 minutes, and the interval of 120 second is 3 second as 1 cycle, a total of 6 cycles (Fig. 1). After pulsed radiofrequency treatment, 2 mL of 10 mg/mL platelet-rich plasma was injected into the joint cavity, the puncture needle was removed, hemostasis was compressed for 3 minutes, and the puncture point was covered with a sterile dressing. Observe for 20 minutes without adverse reactions, and return to the ward on a flat car. After the operation, he was rested in bed for 24 hours, and then injected platelet-rich plasma 3 times in the outpatient department. The injection site was the knee joint cavity. The puncture site was external or internal knee approach. Each dose was 2 mL, once every 1 week.

The pain score (visual analogue scale, [VAS]), curative effect and satisfaction of patients before and 1, 3, and 6 months after operation were recorded. A score of 0 indicates no pain; a score of 1 to 3 indicates mild pain; a score of 4 to 6 indicates moderate pain; and a score of 7 to 10 indicates severe pain. And observe the patient's functional activity limitation recovery after treatment.

## 5 Outcomes

There was no statistical difference in gender and age of the 4 patients. After treatment, they were followed up at 1 month, 3 months, and 6 months. VAS and WOMAC knee score were used to evaluate the pain and pain at 6 months after the operation. Improvements in daily activity ability, active activity range, muscle strength, etc. The VAS and WOMAC scores of 4 patients were significantly reduced at 1 month after surgery. At 3- and 6-months follow-up, there was no significant increase in pain, and the range of motion of the knee joint was significantly improved after surgery (Table 1). There were no complications such as wound swelling, fluid exudation, fever, nerve injury, and vascular injury in all patients.

**Table 1:** VAS and CMS scores of 4 patients.

Patients(No.)	VAS	CMS
No.1 Before surgery	8	64
Postoperative 1 mo	3	33
Postoperative 3 mo	3	34
Postoperative 6 mo	2	32
No.2 Before surgery	8	68
Postoperative 1 mo	3	36
Postoperative 3 mo	2	35
Postoperative 6 mo	2	33
No.3 Before surgery	9	66
Postoperative 1 mo	4	34
Postoperative 3 mo	3	33
Postoperative 6 mo	2	31
No.4 Before surgery	9	65
Postoperative 1 mo	3	35
Postoperative 3 mo	2	34
Postoperative 6 mo	2	35

## 6 Discussion

Knee osteoarthritis has a greater impact on patients and families.^[19,20]^ The visual analogue scoring method was used to evaluate the severity of pain and the objective imaging examination was used as the diagnostic criterion. Among them, Kellgren-Lawrence (K-L) classification is used as the basic standard for X-ray film performance, and Recht classification is used as a supplementary standard for knee MRI performance. Combining the 4 clinical symptoms and signs of knee joint pain, mobility, swelling and deformity, KOA is It is divided into 4 phases, and the corresponding four-step treatment strategy is proposed.^[21]^ Individualized treatment is one of the future medical development trends. The choice of treatment methods should consider factors such as the patient's age, clinical manifestations and personal expectations, adhere to the treatment goal of “precise knee protection,” and implement standardized and standardized treatment methods. Patients tailor the best treatment strategy to maximize the patient's clinical efficacy. In this study, our application of ultrasound-guided pulsed radiofrequency combined with platelet-rich plasma technology to treat knee osteoarthritis is the first case, and there is no similar report.

Intra-articular glucocorticoid injection is often used as the main treatment for knee osteoarthritis, but there is controversy about the degree and duration of symptom relief by this therapy.^[22–24]^ Complications of its injection include joint infections, accelerated degradation of articular cartilage, and subchondral insufficiency fractures.^[25–27]^ And according to the latest research progress, the absolute score of physical therapy for knee osteoarthritis is better than glucocorticoid injection in terms of pain and physiological function.^[28]^ Hyaluronic acid is an acidic mucopolysaccharide naturally present in synovial fluid with elastic viscosity.^[29]^ Because of its lubrication and shock absorption effect on articular cartilage, intra-articular injection of hyaluronic acid is a real treatment method to improve pain and function.^[30]^ However, the Meta analysis of Han^[31]^ and others showed that platelet-rich plasma has better long-term effects than hyaluronic acid in the treatment of knee osteoarthritis, and can relieve pain, improve function, and improve quality of life. The purpose of the combined application of pulsed radiofrequency and platelet-rich plasma therapy is to make full use of the analgesic effect of pulsed radiofrequency technology and the repairing effect of platelet-rich plasma, which can maximize the advantages of the 2 more advanced treatment methods with minimally invasive techniques. The principle of pulsed radio frequency is mainly to produce a rapidly changing strong electric field around the tip of the radio frequency trocar, which can lead to changes in pain signals and pain relief, but the repair effect is not as good as other conservative treatments. The platelet-rich plasma promotes the repair and regeneration of local microenvironmental tissues by releasing platelet-derived growth factor, transforming growth factor-β, and vascular endothelial growth factor.

## 7 Conclusion

The combined application of pulsed radiofrequency and platelet-rich plasma can better promote the improvement of knee osteoarthritis symptoms and shorten the course of treatment, so that patients can restore knee joint function in the shortest time.

## Author contributions

**Conceptualization:** Hui Jin, Hao Zuo, Rui Xu, Youbo Ji, Zhonghan Wang.

**Data curation:** Hui Jin, Hao Zuo, Rui Xu, Youbo Ji, Zhonghan Wang.

**Formal analysis:** Hui Jin, Hao Zuo, Rui Xu, Youbo Ji, Zhonghan Wang.

**Funding acquisition:** Hui Jin, Hao Zuo, Rui Xu, Youbo Ji, Zhonghan Wang.

**Investigation:** Hui Jin, Hao Zuo, Rui Xu, Youbo Ji, Zhonghan Wang.

**Methodology:** Hui Jin, Hao Zuo, Rui Xu, Youbo Ji, Zhonghan Wang.

**Project administration:** Hui Jin, Hao Zuo, Rui Xu, Youbo Ji, Zhonghan Wang.

**Resources:** Hui Jin, Hao Zuo, Rui Xu, Youbo Ji, Zhonghan Wang.

**Software:** Hui Jin, Hao Zuo, Rui Xu, Youbo Ji, Zhonghan Wang.

**Supervision:** Hui Jin, Hao Zuo, Rui Xu, Youbo Ji, Zhonghan Wang.

**Validation:** Hui Jin, Hao Zuo, Rui Xu, Youbo Ji, Zhonghan Wang.

**Visualization:** Hui Jin, Hao Zuo, Rui Xu, Youbo Ji, Zhonghan Wang.

**Writing – original draft:** Hui Jin, Hao Zuo, Rui Xu, Youbo Ji, Zhonghan Wang.

**Writing – review & editing:** Hui Jin, Hao Zuo, Rui Xu, Youbo Ji, Zhonghan Wang.
